# POC1A induces epithelial–mesenchymal transition to promote growth and metastasis through the STAT3 signaling pathway in triple-negative breast cancer

**DOI:** 10.1186/s10020-025-01315-1

**Published:** 2025-08-19

**Authors:** Yuzhou Qian, Yu Che, Shanqi Li, Xue Zhang, Qingshu Li, Yong Zhu, Long Wang, Xuedong Yin

**Affiliations:** 1 Institute of Life Sciences, Chongqing Medical University, Chongqing, 400016 China; 2 Department of Breast and Thyroid Surgery, Chongqing Key Laboratory of Molecular Oncology and Epigenetics, The First Affiliated Hospital of Chongqing Medical University, Chongqing, 400016 China; 3 Department Of Breast Cancer Center, Chongqing University Cancer Hospital, Chongqing, 400030 China; 4 Department of Respiratory Medicine, The First Affiliated Hospital of Chongqing Medical University, Chongqing, 400016 China; 5 Department of Pathology, College of Basic Medicine, Chongqing Medical University, Chongqing, 400016 China; 6 Molecular Medicine Diagnostic and Testing Center, Chongqing Medical University, Chongqing, 400016 China; 7 Department of Pathology, the First Affiliated Hospital of Chongqing Medical University, Chongqing, 400016 China

**Keywords:** POC1A, TNBC, EMT, Metastasis, STAT3

## Abstract

**Objectives:**

Triple-negative breast cancer (TNBC) is known for its aggressiveness, which can be attributed to its heterogeneity, metastasis, and invasion capabilities. POC1 centriolar protein homolog A (POC1A), a centriolar protein involved in the formation of stable centrioles, has been associated with both cancer promotion and suppression in various malignant tumors. However, the underlying mechanisms that drive POC1A-induced metastases in TNBC remain to be elucidated.

**Methods:**

The expression of POC1A changes and their clinical significance have been evaluated using TNBC tissues and a database. POC1A expression was examined in clinical samples and cells. The impacts of POC1A on the epithelial–mesenchymal transition's (EMT) relative factor expression was examined using immunofluorescence (IF), transcription-quantitative PCR (RT-qPCR), and Western blotting. We investigated the migration and invasion capabilities of TNBC cells and found that the patterns of tumor growth and metastasis varied correspondingly in different xenograft models. RNA sequencing (RNA-seq) was performed to explore the signaling pathways involved in POC1A, which was verified by several experiments.

**Results:**

Our study identified an increase in the expression of POC1A in TNBC tissues, which was found to correlate with tumor size and lymph node metastasis. Meanwhile, POC1A plays a crucial role in the process of EMT, regulating the invasion and metastasis of TNBC in vitro and in vivo. Our RNA sequence results, followed by further investigation, revealed that POC1A promotes the metastasis of TNBC by inducing EMT through the STAT3 signaling pathway.

**Conclusions:**

In short, for the first time, we have identified that POC1A plays a pivotal role in regulating the EMT of TNBC.

**Supplementary Information:**

The online version contains supplementary material available at 10.1186/s10020-025-01315-1.

## Introduction

Global cancer statistics for 2023 indicate that the incidence rate of female breast cancer ranked first place while the fatality rate ranked second place (Siegel et al. 2023). Breast cancer is a serious threat to the health of women. According to research, breast cancer patients already have metastases in 3%−10% of cases, while about three-quarters of incipient breast cancer patients progress to end-stage breast cancer (Chen et al. 2016). Notably, TNBC represents a significant proportion of breast cancer cases and is marked by its aggressive nature, high rates of distant metastases, and poor survival outcomes (Bianchini et al. 2022). Ninety percent of breast cancer deaths are metastasis-driven, leading to resistance to treatment (Xie et al. 2019). Therefore, the prognosis of breast cancer patients is primarily affected by metastasis. Understanding its mechanisms can provide novel therapeutic approaches for breast cancer.

EMT is an essential process observed in normal embryonic development and tissue regeneration. However, the abnormal reactivation of EMT has been linked to tumor progression and metastasis, including enhanced migratory and invasive capabilities (Aiello and Kang 2019). EMT is a reversible process wherein epithelial cells undergo a transformation into mesenchymal cells, accompanied by alterations in the cell adhesion molecules and cytoskeletal components. Cancer cells undergoing EMT display morphological and molecular modifications (Huang et al. 2022; Page et al. 2019). This is evidenced by the downregulation of epithelial markers, including E-cadherin, and the upregulation of mesenchymal markers, such as N-cadherin and Vimentin. Furthermore, the pivotal EMT processes are mediated by Snail, Zeb, Twist, etc. (Mittal 2018; Saitoh 2023; Tang et al. 2020; Yang et al. 2013). The regulation of EMT involves various signaling pathways, such as transforming growth factor (TGF), Notch pathways, and the JAK/STAT signaling pathway (Said and Williams 2011; Jin 2020). Among them, the JAK/STAT3 signaling pathway led to increased tumorigenic and metastatic abilities, as well as chemoresistance in cancer, through its enhancement of the EMT (Jin 2020). STAT3 proteins involve tumor oncogenesis. STAT3 is the most extensively studied transcription factor in EMT programs with respect to the progression of tumors. Additionally, STAT3 is known to play a key role in tumor immunity, while other STAT family members are linked to the promotion of cancer development (Yu et al. 2009; Ma et al. 2020). The activation of STAT3 has been found to enhance metastasis by inducing EMT (Sullivan et al. 2009; Yadav et al. 2011; Xiong et al. 2012). PIM1, MCL1, TIMP1 and TIMP2, as downstream target genes of the STAT3 signaling pathway, play an important role in the occurrence and development of tumors. STAT3 binds to promoter of downstream target genes and upregulates their transcription, and plays an indispensable role in regulating cell proliferation, cell cycle, and migration(Mahata et al. 2022; Brault et al. 2010; Zeng et al. 2022; Tian et al. 2022; Escalona et al. 2020). Numerous studies have demonstrated that STAT3 can effectively promote cancer progression in TNBC (Wang et al. 2018). At the same time, the inhibition of STAT3 has been shown to inhibit the development of cancer, invasion, and migration by inhibiting EMT processes. Consequently, STAT3 is now considered to serve as a biomarker for TNBC diagnosis and as a target for treatment intervention (Qi et al. 2023).

POC1A serves as a crucial part of the centrosome, playing a pivotal role in the assembly and stability of centrioles (Koparir et al. 2015; Venoux et al. 2013; Shaheen et al. 2012). Furthermore, POC1A engages in the various functions of the centriole, including centriole duplication and centriole length control, ensuring the integrity of centrioles and the formation of the mitotic spindle (Venoux et al. 2013; Giorgio et al. 2017). Extensive research has established a correlation between POC1A and facial dysmorphism and short stature, all of which are manifestations of aberrant cell mitosis (Koparir et al. 2015; Majore et al. 2021; Ko et al. 2016). According to the findings, it is suggested that POC1A could produce a significant effect on cell proliferation. Thus, it established its role as a cell cycle regulator (Zhao et al. 2022). Currently, POC1A has been studied for its role in tumorigenesis. In a study by Wada et al., POC1A was shown to predict the recurrence of intrahepatic cholangiocarcinoma (Wada et al. 2021). It has been suggested that POC1A may be a target for cancer therapies (Dastsooz et al. 2019). Researchers suggested that POC1A is a valuable biomarker in pan-cancer (Zhao et al. 2022), whereas the impact of POC1A on EMT and its function in the development of TNBC remains uncertain.

Thus, our study mainly explores the effect of POC1A in TNBC. Our findings demonstrate that POC1A facilitates the metastasis and invasion of TNBC cells via the JAK/STAT3 pathway by inducing STAT3-mediated transcriptional activation via the phosphorylation of STAT3. Based on the findings of this study, POC1A is considered to be an oncogene in triple-negative breast cancer.

## Methods

### Patients and datasets

A comprehensive collection of breast cancer RNA expression data was obtained from multiple sources. Specifically, data from the Cancer Genome Atlas (TCGA) cohort (http://portal.gdc.cancer.gov) and an independent cohort, GSE21653, from the Gene Expression Omnibus (GEO) (http://www.ncbi.nlm.nih.gov/geo) were obtained. It is important to note that all data used in our paper are publicly available and abide by the data access policies set forth by TCGA and GEO. Furthermore, we collected human TNBC samples and matched non-tumor tissues from the First Affiliated Hospital of Chongqing Medical University with the explicit consent of each patient. Human specimens will be collected adhere to the University's Institutional Ethics Committee.

### Immunohistochemistry (IHC)

Human TNBC samples and matched non-tumor tissue samples were acquired from the First Affiliated Hospital of Chongqing Medical University for the purpose of IHC staining. This study was conducted following the Institutional Ethics Committees of the First Affiliated Hospital of Chongqing Medical University. IHC was performed using the anti-POC1A antibody (1:100, PA5-110,190, Thermo Fisher). The mouse IHC was performed to analyze the expression of POC1A, N-cadherin, and Vimentin with an anti-POC1A antibody (1:100, PA5-110,190, Thermo Fisher), anti-N-cadherin antibody (1:100, 66,219–1-Ig, Proteintech), and anti-Vimentin antibody (1:100, 60,330–1-Ig, Proteintech). The stained tissues were evaluated by a clinical pathologist. Each sample was scored according to staining intensity (0 = no staining; 1 = weak staining; 2 = moderate staining; 3 = strong staining) and stained cell proportion (1 = 1%−25%; 2 = 25%−50%; 3 = 50%−75%; 4 = 75%−100%).

### Cell lines

In this paper, we utilized six human breast cancer cell lines, namely BT-549, MCF-7, MDA-MB-231, MDA-MB-468, SK-BR-3, and ZR-75–1. MDA-MB-231, MDA-MB-468 and BT-549 are TNBC cell. MCF-7 and ZR-75–1 fell to Luminal A subtype. And SK-BR-3 fell to HER2 over-expression subtype. Additionally, we used the healthy breast epithelial cell line MCF10A. We also used a mouse breast cancer cell line, namely 4T1. All cell lines were purchased by the Chinese Academy of Sciences (Shanghai, China). BT-549, MDA-MB-231, MDA-MB-468, ZR-75–1 and 4T1 were grown in PRMI-1640 medium (Gibco, Thermo Fisher Scientific, China) supplemented with 10% fetal bovine serum (FBS) (Gibco, Thermo Fisher Scientific, China), 1% streptomycin, and penicillin. MCF-7 and SK-BR-3 were maintained in DMEM High Glucose with 10% FBS (Gibco, Thermo Fisher Scientific, China), 1% streptomycin, and penicillin. MCF10A was cultured in DMEM/F12 medium and contained 10% FBS, 1% penicillin, and streptomycin. All cells were maintained at 37 °C with 5% CO2.

### RT-qPCR

We extracted all RNAs as previously described (Lambert et al. 2017). A normalized GAPDH was used to quantify the relative expression of POC1A mRNA. Supplementary Table 1 lists the primer sequences.

### Stable knockdown of POC1A

MDA-MB-231 and BT-549 cells were infected with lentiviral vectors shPOC1A#1 or shPOC1A#2 and Scramble. The transfection process involved the use of Lipofectamine2000 (Invitrogen, Thermo Fisher Scientific, China) as the transfection reagent, followed by treatment with 1ug/ml puromycin (Solarbio, P8230, China) in the medium for a duration of two weeks to establish stable POC1A knockdown cells. Primer sequences are shown in Supplementary Table 1.

### Cell viability assay

The cell counting kit 8 (CCK-8) method is a highly sensitive, non radioactive colorimetric detection method used to determine the number of live cells in cell proliferation or toxicity experiments. The 2000 cells were placed in 96-well plates. We used the CCK-8 (Beyotime, Jiangsu, China) reagent to detect cell viability at 24-, 48-, and 72-h intervals. Optical density (OD) values should be measured at 450 nm using a microplate reader (Bench markPlus™ system, Bio‑Rad Laboratories, Inc.) at the specified time points. Accuracy and reproducibility were ensured by repeating each step.

### Colony formation experiment

We placed 800 cells in a 6-well plate with 2 ml PRMI-1640 medium (Gibco, Thermo Fisher Scientific, China) supplemented with 10% fetal bovine serum (FBS) (Gibco, Thermo Fisher Scientific, China), 1% streptomycin, and penicillin. After two weeks, the cells were fixed with 4% paraformaldehyde and stained with crystal violet (C0121, Beyotime). Visible colonies were counted in OLYMPUS microscope (BX53F2).

### Western blotting

Bicinchoninic Acid Assays (Beyotime, Shanghai, China) were used to determine protein concentrations. After denaturation via heating, 10% sulfate–polyacrylamide gel electrophoresis (SDS–PAGE) was carried out on proteins, and then they were transferred to polyvinylidene fluoride (PVDF) membranes for detection using the standard Western blotting procedure. Supplementary Table 2 shows the antibodies.

### Cell cycle analysis

Cells were collected 2 days after planting in 6-well plates (1.0 × 10^6^ cells/well), washed on the 3rd day with phosphate-buffered saline (PBS), and fixed 24 h with 75% ethanol at −20 °C. After being stained with propidium iodide (PI), cells were analyzed using a flow cytometer (FACSVerse, BD, USA) from the Institute of Life Sciences, Chongqing Medical University.

### Migration and invasion assay

The invasion of MDA-MB-231 and BT-549 cells was detected using Transwell chambers with matrigel. The upper chamber added 6 × 10^4^ cells in 150 μl of serum-free medium. In total, 500 μl of a medium containing 10% FBS was added into the lower chamber. The plates were then incubated in a 5% CO2 environment at 37 °C for 48 h. Following incubation, the cells were fixed with 4% paraformaldehyde for 30 min and stained with crystal violet for 1 h (C0121, Beyotime). Then, the stained cells were captured OLYMPUS microscope (BX53F2) and counted for five of fields per membrane that are imaged (Scale bars:200um).

### Cell wound-healing assay

Cell migration was assessed in a wound-healing assay. Cells were plated on 6-well plates and incubated for 0, 24 after wound scratching. Wound confluence was captured at different time points using OLYMPUS microscope (BX53F2). Wound area was caculated using ImageJ by comparing the mean relative wound density of three replicates.

### Immunofluorescence

We performed IF following our study (Wang et al. 2023). MDA-MB-231 and BT-549 cells were incubated with anti-Vimentin (1:500, Proteintech, 60,330–1-Ig), followed by the secondary antibody (1:500, Proteintech, SA00013-3). DNA was stained using DAPI (DAPI, Beyotime, P0131). Imagines were obtained under an OLYMPUS fluorescence microscope (BX53F2).

### RNA-sequence (RNA-seq)

The global gene expression profiles of POC1A knockdown cell and control cells were examined by RNA-seq in SeqHealth (Wuhan, China), and then the Enrichr tool was used to analyze the biological process.

### Animal experiment

In our study, female BALB/c mice aged five weeks were purchased from the Byrness Weil biotech Ltd. For the xenografts, a total of 2.0 × 10^5^ 4T1 cells were suspended in 100ul 1 × PBS and were implanted subcutaneously into the right flanks of BALB/c mice. For the lung metastasis model, the cells were implanted into the tail vein. We measured tumor length and width every 4 days and calculated tumor volumes using the formula of length × width^2^/2. After 20 days, tumor weights were calculated after the mice were euthanized. Tumor tissues and lung tissues were stained for HE (hematoxylin–eosin) staining. All animal experiments adhered to the Animal Ethics Committee of Chongqing Medical University.

### Statistical analysis

The results, as depicted in the figure, were obtained from distinct samples and encompassed all available data. The data are presented as means ± standard deviation (SD) from a minimum of three independent experiments. Statistical analysis was performed using two-tailed unpaired or paired t-tests, as well as one-way or two-way analysis of variance (ANOVA) as appropriate, and a *P* value of < 0.05 represents statistical significance.

## Results

### POC1A is upregulated in TNBC

**Fig. 1:**
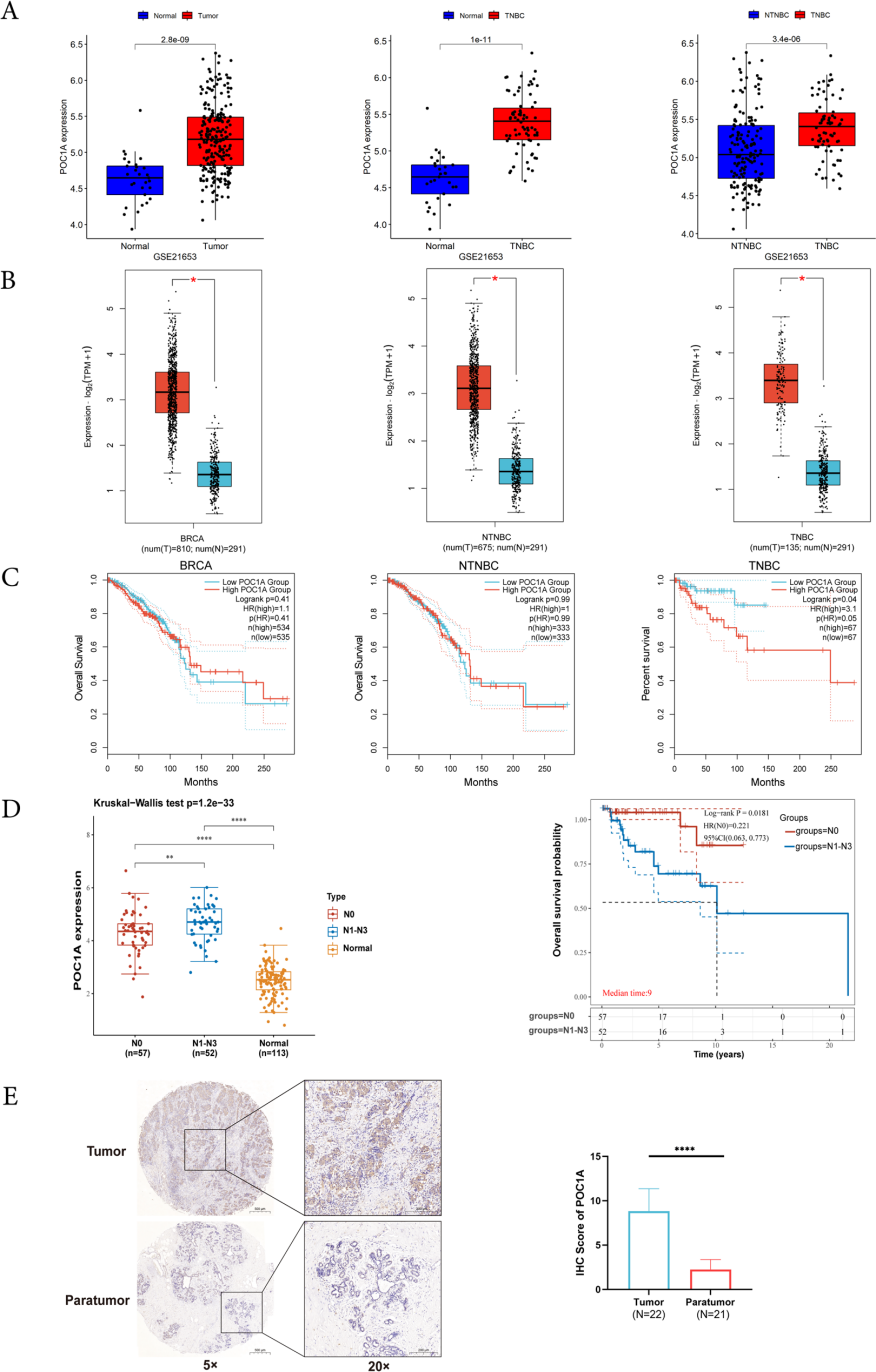
The expression levels of POC1A in TNBC tissues. **A** POC1A mRNA expression levels in GSE21653 datasets from GEO databases in various subgroups of patients with breast cancer. **B** Examination of POC1A expression in various TCGA database subtypes of patients with breast cancer. **C** The TCGA-BRCA cohort's Kaplan–Meier survival study of POC1A. **D** In the TCGA database, the POC1A expression levels and overall survival between the lymph node metastasis group and the non-lymph node metastatic group. **E** POC1A immunohistochemistry staining in TNBC tissues and adjacent non-tumor tissues, together with the H-score values for the two groups. The human TNBC samples and matched non-tumor tissues from the First Affiliated Hospital of Chongqing Medical University. *P* values were computed using unpaired, two-tailed Student's t-tests in **A**, **B**, and **D**. Student's t-tests, two-tailed and paired, were used to determine the P value in **E**. Abbreviations: TNBC: triple-negative breast cancer; GEO: Gene Expression Omnibus; IHC: immunohistochemistry; TCGA: The Cancer Genome Atlas; ****, *p* < 0.0001. The number of experiments in parallel (*n* = 3)

Initially, we proved through the Kaplan Meier database (http://kmplot.com/analysis) that increased POC1A expression was related to reduced overall survival (OS), disease-free survival (DFS), and recurrence-free survival (RFS) in breast cancer patients (Supplementary Fig. 1). In addition, we obtained information from the public GEO-Breast Cancer (BRCA) database. Moreover, our analysis of GSE data confirmed higher POC1A expression in breast cancer tissues in GSE21653, particularly in patients with TNBC (Fig. 1A). In addition, the results are consistent with the above GSE data (Fig. 1B). The survival analysis demonstrated that elevated POC1A expression was obviously linked with poor breast cancer prognosis in TNBC patients (Fig. 1C). We also analyzed the TNBC patient clinical follow-up information from the TCGA database. The mean value of POC1A expression was used as the key threshold. POC1A expression was positively related to age, lymph node metastasis, and pathologic stage (Table 1). The results exhibited that the POC1A had higher expression in TNBC patients with lymph node metastasis compared to TNBC patients without lymph node metastasis. In TNBC patients with lymph node metastasis, overall survival (OS) was the shortest (Fig. 1D). Then, we estimated the POC1A protein expression level in TNBC tissue chips and discovered that POC1A expression was upregulated (Fig. 1E). Thus, it can be observed that POC1A expression is obviously increased in TNBC, which is linked to the prognosis and pathological features of breast cancer patients.

**Table 1:** Clinicopathological features of TCGA triple-negative breast cancer patients

Characteristic	Number of Cases	POC1A		
		High (n)	Low (n)	*P*-value
Age				
> 50	102	44	58	0.04*
≤ 50	66	40	26	
Pathologic stage				
I	25	9	16	0.0174*
II	105	61	44	
III	33	12	21	
IV	3	0	3	
Unknown	2	2	0	
Tumor Size				
T1	42	20	22	0.6416
T2	98	52	46	
T3	18	8	10	
T4	9	3	6	
Unknown	1	1	0	
Lymph node metastasis				
N				
N0	97	44	53	0.005*
N1	45	32	13	
N2	18	6	12	
N3	8	2	6	
Metastasis				
M				
M0	142	70	72	0.6513
M1	4	1	3	
Unknown	22	13	9	

*Represent statistically significant

### POC1A promotes the proliferation of TNBC cells

**Fig. 2:**
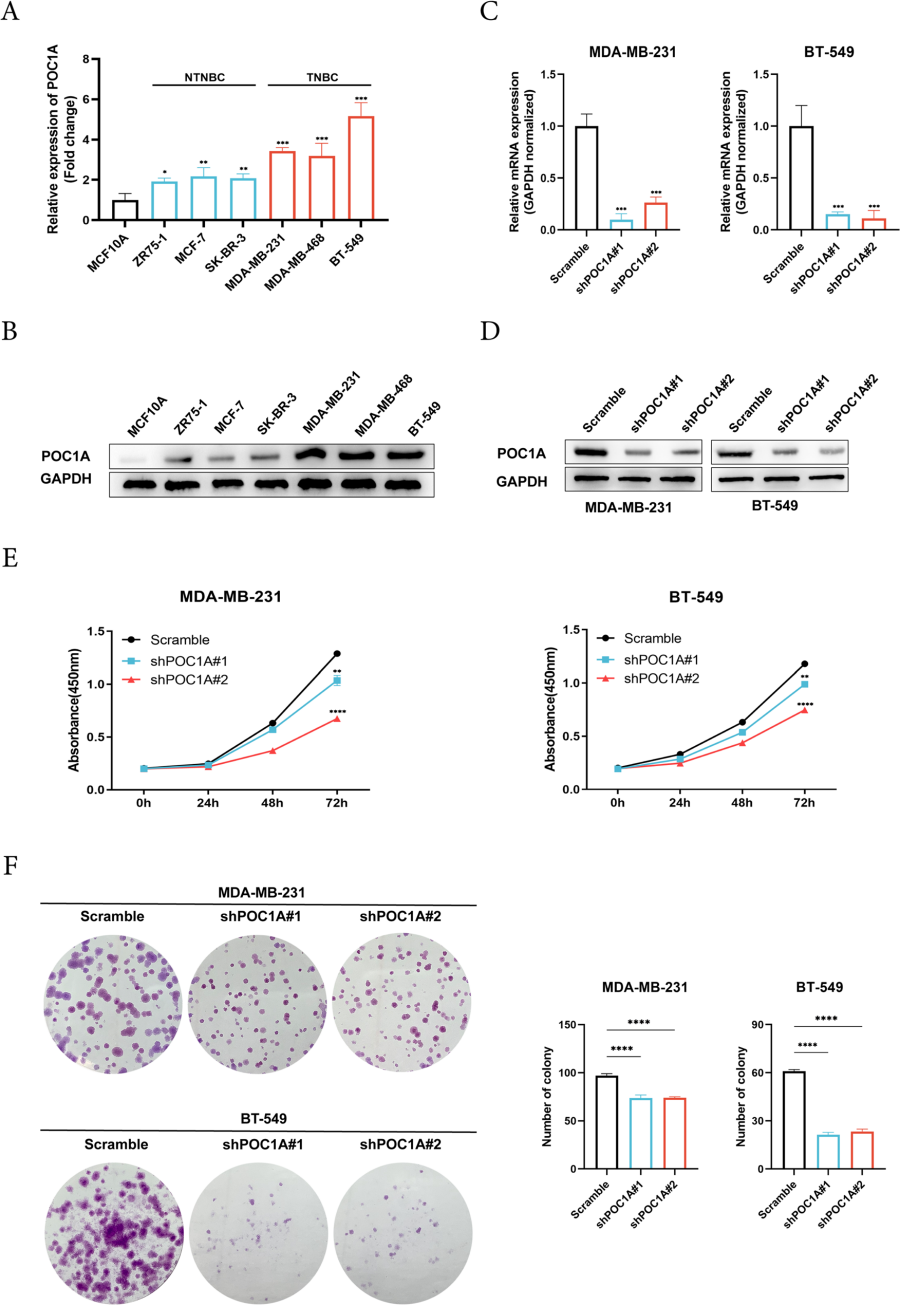
POC1A promotes the proliferation of TNBC cells in vitro. **A**-**B**. RT-qPCR and Western blotting were used to investigate the expression of POC1A mRNA. **A** and protein (**B**) in seven different types of cells. GAPDH was used as the loading control and indicate the intensity ratio of each band. C-D. Lentiviral vectors encoding POC1A short hairpin RNA vectors (shPOC1A#1 or shPOC1A#2) or Scramble (Scr) vectors were used for transfection into MDA-MB-231 and BT-549 cells. POC1A expression was determined via RT-qPCR (**C**) and Western blotting (**D**). **E** The CCK-8 assay determined the viability of POC1A knockdown in MDA-MB-231 and BT-549 cells after 0 h, 24 h, 48 h, and 72 h. **F** The colony-forming test assessed the capacity of POC1A knockdown cells to proliferate in MDA-MB-231 and BT-549 cells. The number of colony cells is presented as the mean ± SD of three independent experiments. All experiments were repeated three times independently with similar results. Data represent mean ± SD. Statistical significance was determined via a two-tailed unpaired t-test. Abbreviations: *, *p* < 0.05; **, *p* < 0.01; ***, *p* < 0.001; ****, *p* < 0.0001. The number of experiments in parallel (*n* = 3)

To explore the biological functions of POC1A in TNBC proliferation, the expression of POC1A in various breast cancer cell lines was evidenced. Our findings revealed a significant elevation of POC1A expression in breast cancer cells compared to MCF10A, with the highest expression observed in TNBC (Figs. 2A-2B). Stable clones were established with shPOC1A plasmids in MDA-MB-231/BT-549 cells, and verification was conducted. Initially, we evaluated the impact of POC1A on TNBC cell proliferation, and the findings from the CCK8 assay and colony formation experiment displayed a significant reduction in proliferation following POC1A silencing (Fig. 2E-2F).

### POC1A arrests the G2/M phase and regulates the migration and invasion of TNBC cells

**Fig. 3:**
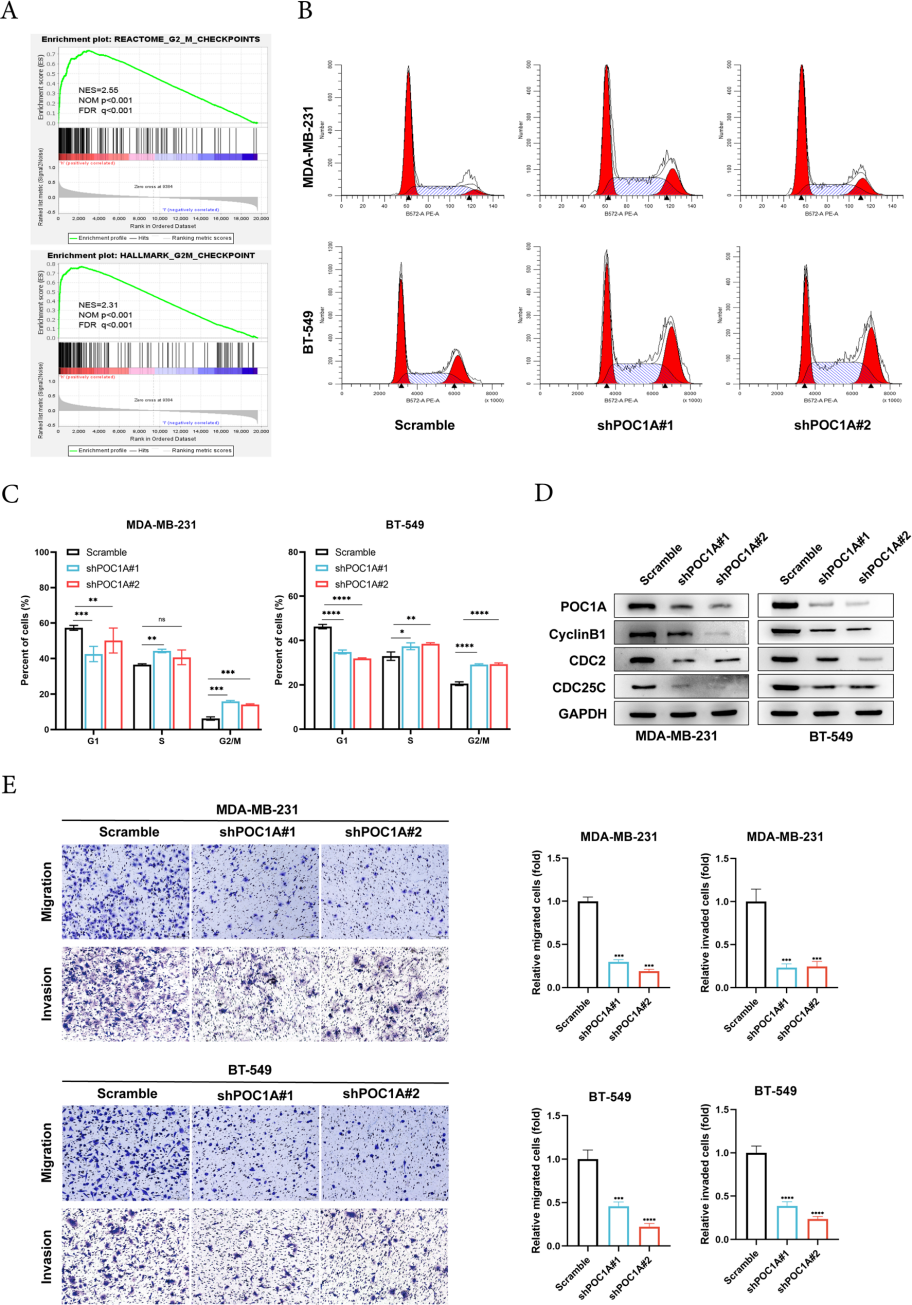
POC1A induces G2/M phase transition and regulates the migration and invasion of TNBC cells. **A** Gene enrichment plots revealed that the POC1A-high subgroup was enriched in a number of gene sets, including Reactome G2 M checkpoints and Hallmark G2 M checkpoints (cell cycle). **B** The cell cycles of POC1A knockdown cells in MDA-MB-231 and BT-549 cells were studied using flow cytometry. **C** Statistical plots show the percentage of each group cell in the G1, S, and G2/M stages. **D** Western blot analysis was used to evaluate the expression of cell-cycle-related proteins in POC1A-knockdown cells. GAPDH was used as the loading control and indicate the intensity ratio of each band. **E** The transwell assay was used to assess cell migration and invasion. The number of cells that moved or invaded was provided as the mean SD of three independent tests. All experiments were independently replicated three times with identical findings. The number of migrated or invaded cells is presented as the mean ± SD of three independent experiments. All trials were carried out three times in a row, with identical results. Data represent mean ± SD. The two-tailed unpaired t-test was used to establish statistical significance. Abbreviations: *, *p* < 0.05; **, *p* < 0.01; ***, *p* < 0.001; ****, *p* < 0.0001. The number of experiments in parallel (*n* = 3)

To identify the effect of POC1A on cell cycle progression, REACTOME and HALLMARK pathways were enriched (Fig. 3A). We detected the impact of POC1A on cell cycles using flow cytometry. The result showed that POC1A knockdown resulted in MDA-MB-231/BT-549 cell cycle arrest at the G2/M phase (Fig. 3B-3C). Furthermore, the downregulation of POC1A leads to cell cycle arrest in the G2/M phase, as evidenced by the decreased expression of key regulators of this phase, including Cyclin B1, CDC2, and CDC25C (Fig. 3D). These findings identified that the downregulation of POC1A inhibits the proliferation of TNBC cells by influencing the cell cycle. In addition, transwell assays were conducted to reveal the impact of POC1A on TNBC cell migration and invasion, revealing that the knockdown of POC1A weakened the metastasis abilities of TNBC cells (Fig. 3E). Collectively, our findings indicated that POC1A results in TNBC migration and invasion in cells.

### POC1A promotes TNBC migration and invasion by regulating EMT

**Fig. 4:**
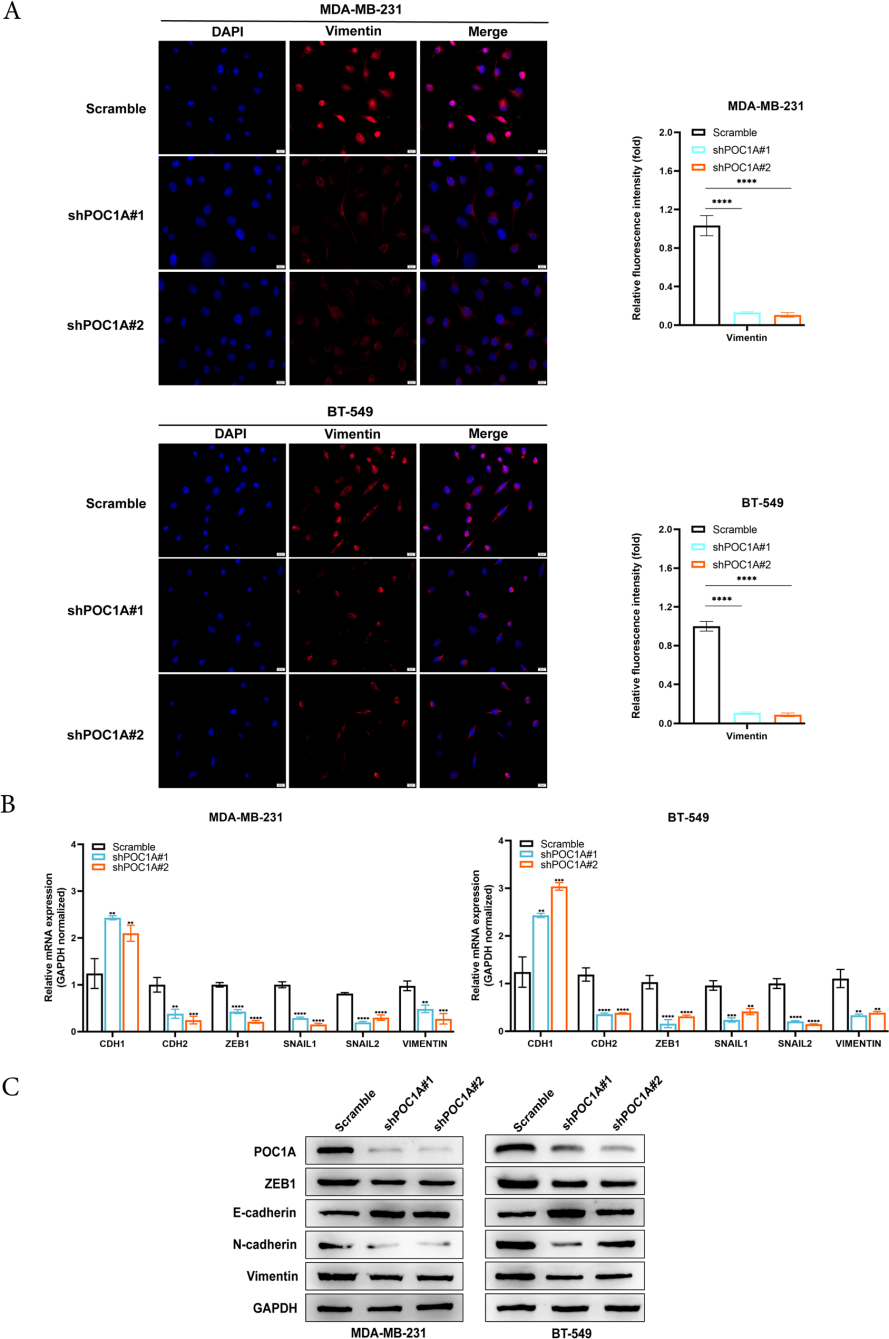
POC1A promotes TNBC metastasis by regulating EMT. **A** Immunofluorescence staining reveals a change in mesenchymal markers in the POC1A knockdown cell clones indicated. **B**-**C** RT-qPCR (**B**) and Western blotting (**C**) show that the key epithelial and mesenchymal markers change in the corresponding POC1A knockdown cell clones. GAPDH was used as the loading control and indicate the intensity ratio of each band. All trials were carried out three times in a row with identical results. The experiments were repeated three times independently, with similar results. Data represent mean ± SD. *P* values in A were calculated using unpaired two-tailed Student’s t-tests. Abbreviations: **, *p* < 0.01; ***, *p* < 0.001; ****, *p* < 0.0001. The number of experiments in parallel (*n* = 3)

Immunofluorescence staining confirmed a decrease in Vimentin expression in cells with POC1A knockdown (Fig. 4A). Meanwhile, RT-qPCR results showed that the silencing of POC1A increased the mRNA expression of CDH1, while the mRNA expression of VIM, CDH2, SNAIL1, SNAIL2, and ZEB1 decreased (Fig. 4B). Moreover, Western blot analysis revealed that the knockdown of POC1A increased E-cadherin protein expression, whereas ZEB1, N-cadherin, and Vimentin protein expression decreased (Fig. 4C). These data demonstrated that POC1A induced EMT in vitro.

### POC1A activates the STAT3 signaling pathway in TNBC cells

**Fig. 5:**
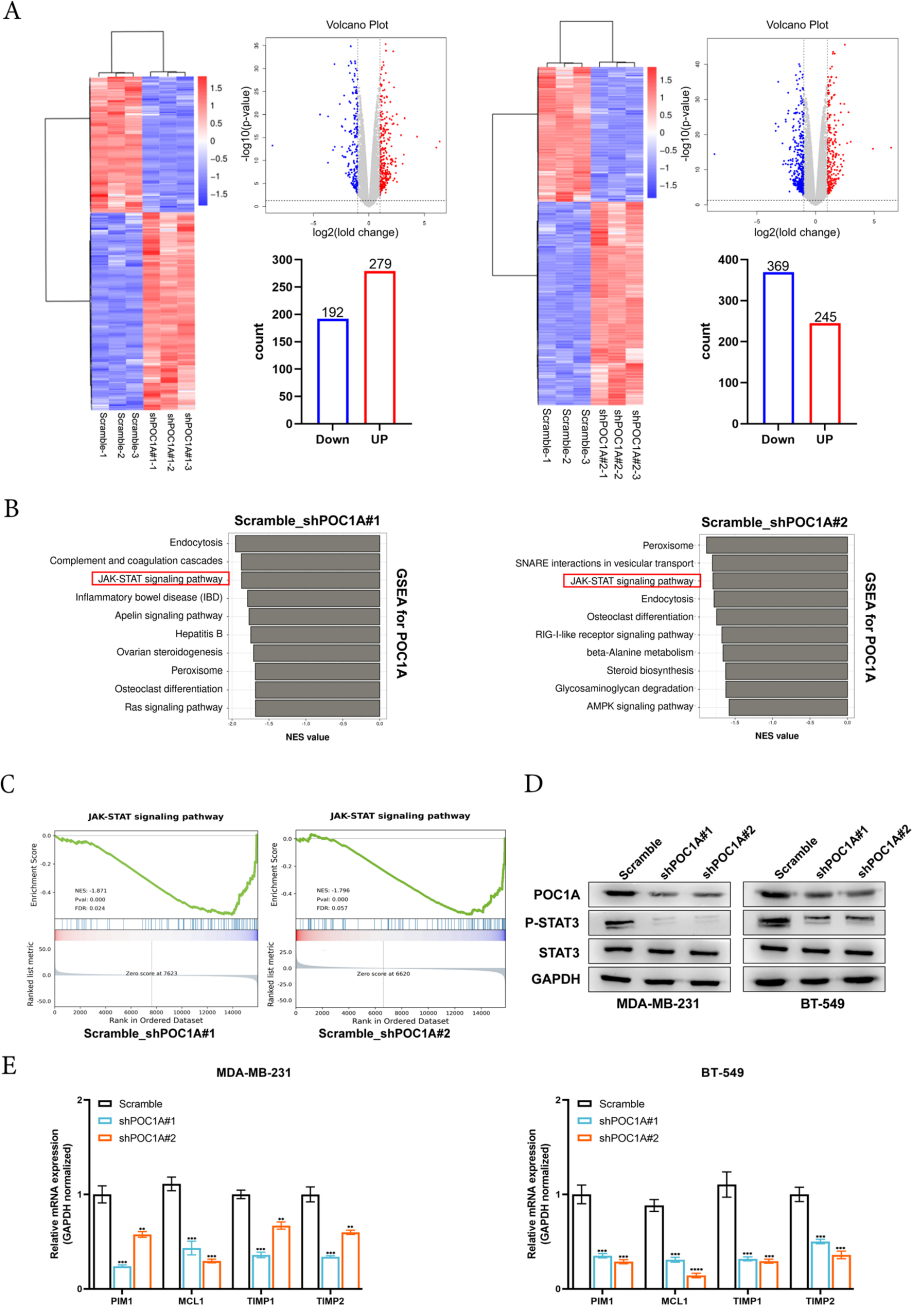
POC1A activates the STAT3 signaling pathway in TNBC. **A** heatmap and volcano plot showing genes that were found to be differentially expressed via RNA-seq in the POC1A knockdown cell (MDA-MB-231). **B** Using RNA-seq data, GSEA analysis revealed POC1A-related pathways in MDA-MB-231 cells. **C** GSEA plots of RNA-seq data from POC1A expression were found to be adversely correlated with JAK-STAT signaling in MDA-MB-231 cells. FDR, false discovery rate; P value; NES, normalized enrichment score. **D** Western blotting was used to identify the expression of p-STAT3 (Tyr705) and STAT3 in POC1A knockdown MDA-MB-231 and BT-549 cells. GAPDH was used as the loading control and indicate the intensity ratio of each band. E. RT-qPCR was used to measure the mRNA level of STAT3 downstream targets. All experiments were repeated three times independently, with similar results. Data represent mean ± SD. *P* values in **E** were calculated using unpaired two-tailed Student’s t-tests. Abbreviation: GSEA: gene set enrichment analysis; **, *p* < 0.01; ***, *p* < 0.001; ****, *p* < 0.0001. The number of experiments in parallel (*n* = 3)

To further investigate the mechanism of POC1A in regulating TNBC progression, POC1A knockdown, and Scramble MDA-MB-231 cells were used to perform RNA-seq. As shown in the heatmaps and volcano plot, 279 genes (dark red dots) were upregulated, and 192 genes (dark green dots) were downregulated in shPOC1A#1 compared to the Scramble group. In total, 245 genes (dark red dots) were upregulated, and 369 genes (dark green dots) were downregulated in shPOC1A#2 compared to the Scramble group in MDA-MB-231 (Fig. 5A). Additionally, based on the mRNA expression profiles of POC1A, GSEA analysis indicated that POC1A was mainly enriched in JAK-STAT signaling pathways (Fig. 5B-5C). Thus, to prove that POC1A produces a significant effect in the STAT3 signaling pathways, the expression of the STAT3 pathway-related proteins, STAT3 and p-STAT3 (Tyr705), was detected via Western blot. The silencing of POC1A decreased the expression of p-STAT3, whereas the protein levels of STAT3 remained static in MDA-MB-231 and BT-549 cells (Fig. 5d). Moreover, the STAT3 pathway regulated the downstream genes, such as PIM1, MCL1, TIMP1, and TIMP2. Our study indicated that the mRNA levels of these genes were downregulated after POC1A knockdown in these cells (Fig. 5E).

### STAT3 facilitates POC1A-induced STAT3 signaling activation in TNBC cells

**Fig. 6:**
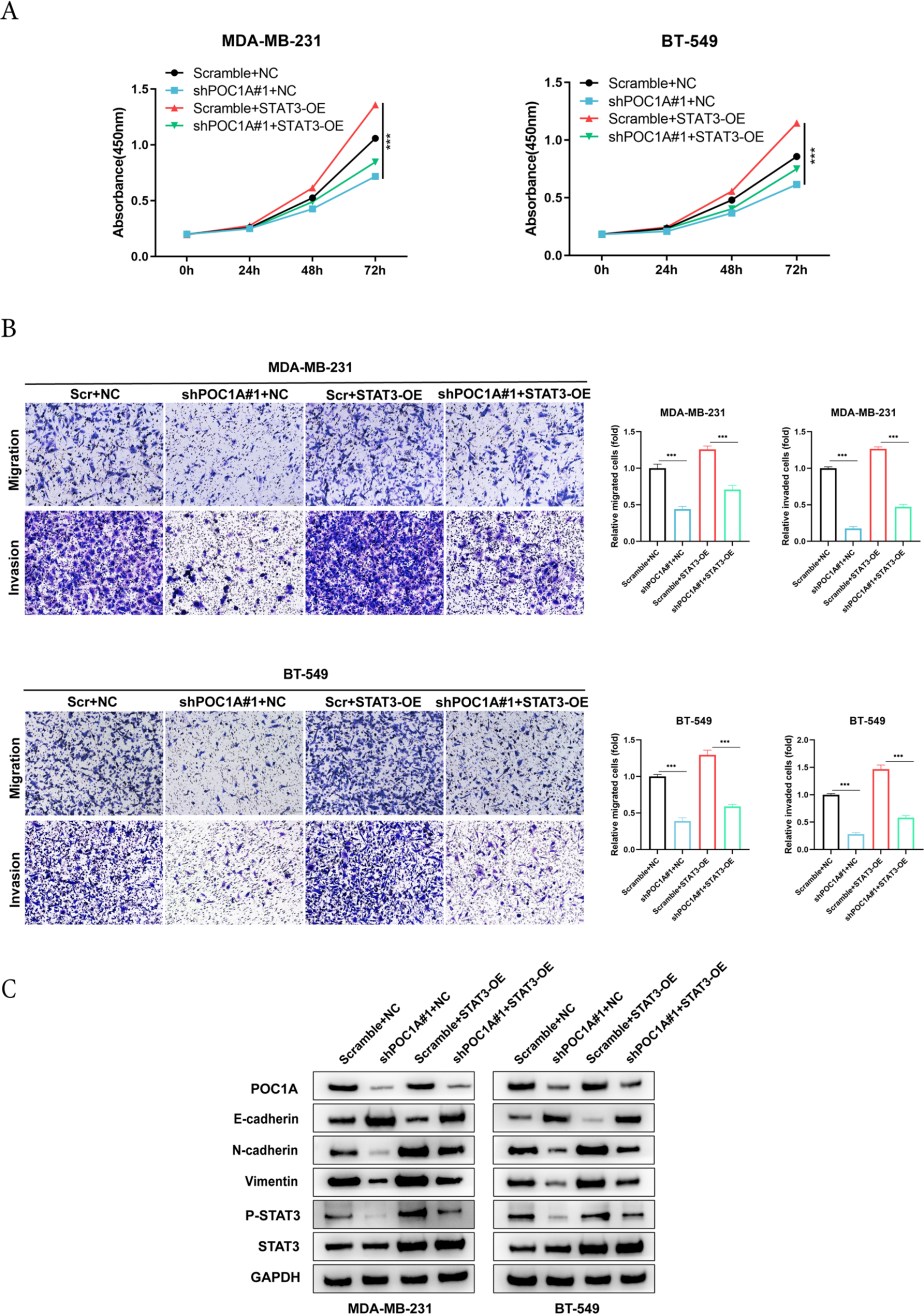
STAT3 facilitates POC1A-induced STAT3 signaling activation in TNBC cells. **A** POC1A knockdown and Scramble cells were transfected with STAT3; the CCK-8 assay indicated that POC1A rescued the proliferation of TNBC cells following the transfection of STAT3. **B** Transwell assay indicated that POC1A promoted the invasion and metastasis of TNBC cells following the transfection of STAT3. **C** Protein levels of POC1A, E-cadherin, N-cadherin, Vimentin, STAT3, and p-STAT3 (Tyr705) were detected via Western blotting. GAPDH was used as the loading control and indicate the intensity ratio of each band. A one-way ANOVA followed by a post hoc Tukey's test was used for comparisons between groups. Abbreviation: ***, *p* < 0.001. The number of experiments in parallel (*n* = 3)

Finally, we explored the effect of STAT3 signaling for POC1A in the regulation of EMT in TNBC cells. STAT3 plasmid was used to reverse the function of POC1A on the proliferation ability of MDA-MB-231 and BT-549 via the CCK8 assay (Fig. 6A). Subsequently, the transwell assay was utilized to observe the impact of STAT3 overexpression on the metastatic capacities of MDA-MB-231 and BT-549 cells (Fig. 6B). The expression of related factors on EMT and the protein of the STAT3 pathway was checked using Western blot. We observed upregulation in the expression levels of Vimentin, N-cadherin, STAT3, and p-STAT3 (Tyr705), while E-cadherin expression was downregulated (Fig. 6C). This finding demonstrates that overexpressing STAT3 increases proliferation, invasion and migration independently of POC1A expression. What this figure shows is that STAT3 overexpression increases P-STAT3 levels and increases expression of N-cadherin and Vimentin while decreases E-cadherin expression, in agreement with EMT activation.

### Silence of POC1A inhibits tumor growth and metastasis of TNBC in vivo

**Fig. 7:**
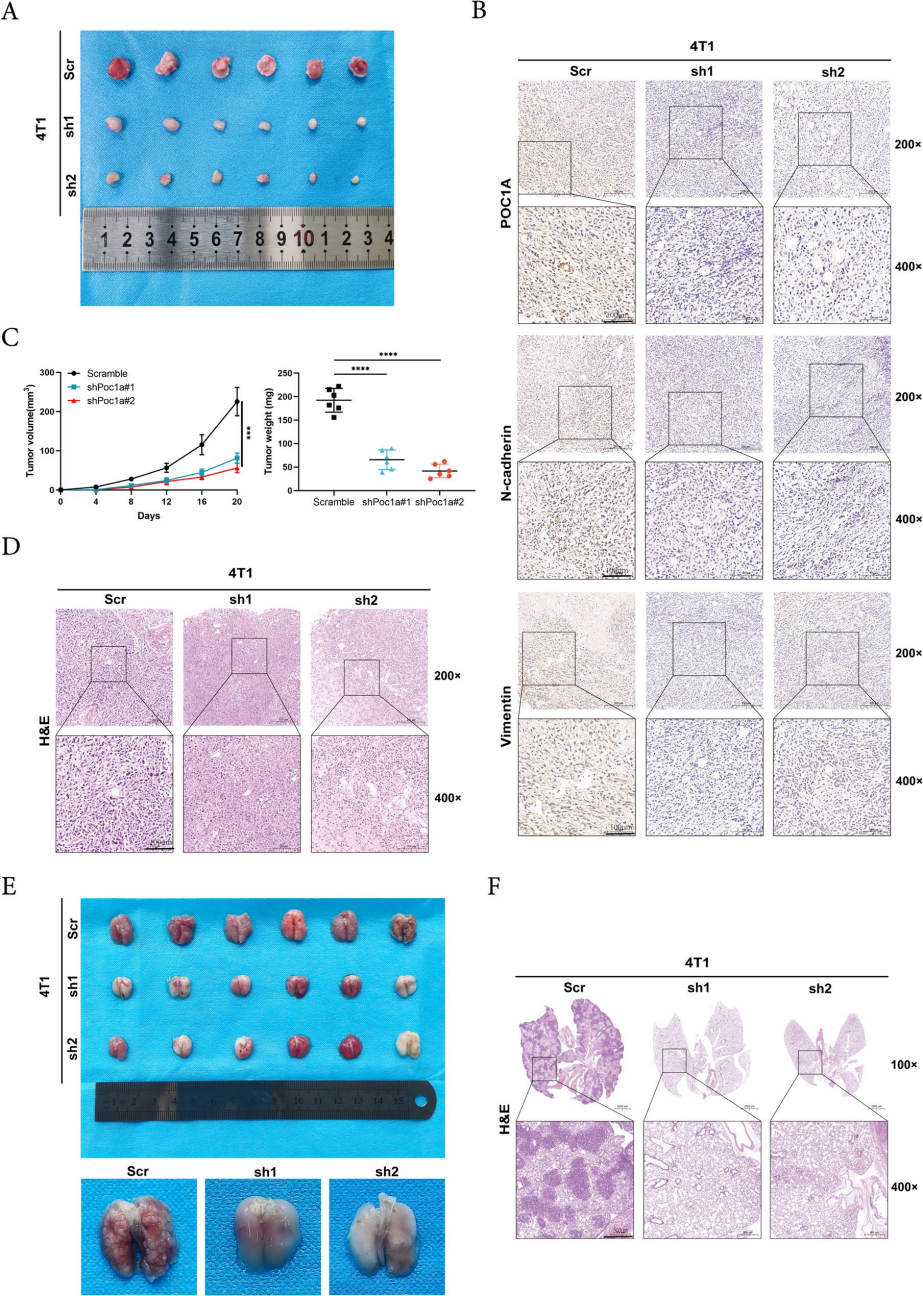
Knockdown of POC1A inhibits tumor growth and metastasis of TNBC in vivo. **A** The silencing of POC1A inhibited subcutaneous tumor formation in the BALB/c mouse model. **B** Tumor volume and weight analysis of subcutaneous tumors in nude mouse models. **C** Typical tumor sample IHC staining results. **D** Sample pictures of HE staining from (**A**), with a 500 µm scale bar. **E**–**F** A representative visualization of lung metastasis models (**E**) and a sample of a lung metastasis specimen stained with HE (**F**). Abbreviation: ****, *p* < 0.0001. The number of experiments in parallel (*n* = 3)

To examine the role of POC1A on tumor growth in vivo, we introduced Poc1a shRNA or Scramble shRNA into 4T1 cells. RT-qPCR and Western blot revealed the silencing efficiency of stable clones, the CCK8 experiment indicated a decrease in proliferation upon 4T1-Poc1a silencing, and wound healing assay showed that 4T1-Poc1a knockdown inhibited the cell's migration ability (Supplementary Fig. 2). The cells were subcutaneously implanted into mice. The BALB/c mice of 4T1-shPoc1a groups grew smaller tumors compared to the Scramble group (Fig. 7A). Next, the tumor size was also obviously smaller in the 4T1-shPoc1a mouse group compared with 4T1-Scramble (Fig. 7B). As is shown in Fig. 7C, our result indicated that the silencing of Poc1a could obviously inhibit the expression of POC1A, N-cadherin, and Vimentin in mice tumor tissues using IHC, and the effect of Poc1a knockdown on the pathology via HE staining is shown (Fig. 7D). To detect metastasis activity, we established a lung metastasis model. The volume and number of metastatic lung foci decreased noticeably by POC1A knockdown, and similar results were verified by HE staining between the control group and the POC1A knockdown group as well (Fig. 7E-F). The above data showed that POC1A inhibited the proliferation and metastasis of TNBC cells in vivo.

## Discussion

Breast cancer is considered one of the most terrible female diseases, and there has been a significant increase in incidence and mortality in recent years. TNBC is the most malignant breast cancer tumor, and it has higher rates of metastases and is more aggressive than other breast cancer subtypes (Huo et al. 2022). Cancer metastasis is a complex process encompassing various crucial stages, including migration, invasion, adhesion, and metastatic colonization (Lambert et al. 2017; Bidard et al. 2008; Wu et al. 2016). In recent decades, the treatment of TNBC patients benefited from the in-depth exploration of TNBC molecular mechanisms, but the underlying mechanism has not been completely explored. A more in-depth exploration of the metastasis mechanisms in TNBC is vital for promoting the prognosis of patients with clinical TNBC and providing guidance for TNBC treatment.

In our study, we collected data from breast cancer patients from the GSE21653 dataset and analyzed the expression state of POC1A between normal tissue and breast cancer tissue. We observed an upregulation of POC1A expression in breast cancer tissues. Then, the data of breast cancer patients were divided into TNBC and NTNBC groups. The expression of POC1A was upregulated significantly compared with the NTNBC group. The same results can be obtained from the TCGA database. Moreover, the high expression of POC1A was associated with poorer TNBC patient OS. Further analyses revealed that POC1A was highly expressed, and poor OS in TNBC was observed in lymph node metastasis patients. The data were matched with a series of clinical features from clinical follow-up information. To confirm the above results, we first analyzed the tissue expression level of POC1A in TNBC cancer, and it was revealed that POC1A levels exhibited a significant increase in TNBC tissues compared to matched tumor tissues. Moreover, consistent results can also be obtained at the mRNA and protein levels. According to these results, we propose that POC1A may become involved in the tumorigenesis of TNBC.

To explore the functions of POC1A, we carried out POC1A knockdown studies in TNBC cells. The results of all experiments displayed that silencing POC1A led to a notable inhibition with respect to proliferation, invasion, and metastasis of TNBC in vitro and in vivo. A positive association was observed in the expression of POC1A and EMT. The acquisition of migratory and invasive capabilities represents the most pivotal steps in metastasis development. Cells experiencing these alterations undergo profound morphological changes, which are collectively known as the EMT process. EMT is widely recognized as a crucial mechanism in regulating cancer metastasis (Majore et al. 2021; Dastsooz et al. 2019). The EMT programs involved in the loss of polarity and the disassembly of epithelial cell–cell contacts, and the cells undergoing EMT are reorganized, permitting mesenchymal morphologies and an increase in motility. Furthermore, cells with EMT features are often able to degrade and invade their basal extracellular matrix (Nieto et al. 2016; Lu and Kang 2019). Several core EMT transcription factors also coordinate this complex program in promoting tumor metastasis. Major EMT-inducing transcription factors include SNAI1 and SNAI2, TWIST1 and TWIST2, and ZEB1 and ZEB2 (Stemmler et al. 2019). SNAI1, as a transcription activator, can induce mesenchymal genes directly (Hsu et al. 2014). In tumors, the expression of SNAI1 and SNAI2 leads to decreased E-cadherin levels and enhanced tumor cell metastasis, and it is associated with the poor prognosis of breast cancer patients (Blanco et al. 2002; Tran et al. 2014). TWIST can downregulate E-cadherin and activate N-cadherin and vimentin, and it is vital for tumor cell dissemination and metastasis in breast cancer (Lamouille et al. 2014; Xu et al. 2017). ZEB1 and ZEB2, are members of the Zeb transcription factor family, and ZEB proteins directly downregulate the expression of tight junction genes to drive EMT (Vandewalle et al. 2005). ZEB1 and ZEB2 have been experimentally shown to promote cell migration and invasion in breast cancer and pancreatic cancer (Spaderna et al. 2008; Krebs et al. 2017). In addition, EMT is also coordinated by multiple other factors and works together. The TGF‑β, Wnt/β-catenin, and NF-κB signaling pathways play an important role in EMT (Xu et al. 2009; Hseu et al. 2019). TGF-β is a strong driver of EMT. TGFβ and their receptors chronically exist in TNBC, and they are related to metastatic phenotypes (Ivanovic et al. 2003). The study reported that ZL170 can inhibit TNBC invasion and metastasis by targeting TGFβ signaling pathways (Di et al. 2019). The activation of Wnt has been shown to play a key effect in regulating EMT via canonical pathways (β-catenin) (Yin et al. 2018). The Wnt pathway also upregulates EMT-related factors like Slug and Twist, and it promotes breast cancer metastasis (Yook et al. 2006). Moreover, some results showed NF-κB signaling in the induction and maintenance of invasive phenotypes linked to EMT (Oh et al. 2023).

In addition to the research mentioned above, our study discovered that POC1A was regulated by the STAT3 pathway in TNBC. Our experimental data showed that silencing of POC1A enabled STAT3 itself to remain static, but it decreased the expression of p-STAT3 (Tyr705) proteins in TNBC cells. Previous studies have shown that centrosome proteins can regulate the assembly of signal molecule complexes through spatial localization. POC1A, as a centrosomal protein, may recruit STAT3 and upstream kinases (such as JAK2) to specific subcellular regions through its centrosomal localization properties, thereby promoting efficient phosphorylation of STAT3. In addition, because POC1A is involved in ciliary formation, it may also indirectly affect STAT3 activity by interfering with Hedgehog or PDGFRα signaling. The STAT family (STATs 1–4, 5α, 5β, and 6) has seven members (STATs 1–4, 5α, 5β, and 6). Each STAT protein has conserved domains (Zhang et al. 1999). The SH2 domains of STAT3 proteins are important for STAT–cytokine receptor interactions as they form stable homo- or heterodimers with other STAT proteins (Hemmann et al. 1996). The tyrosine residue (Tyr705) that is between the SH2 and transactivation domain results in the phosphorylation of STAT3. Then, cytokines induce the dimerization and nuclear translocation of STAT3, and they can regulate the transcription of downstream target genes in the STAT3 signaling pathway (El-Tanani et al. 2022; Chakraborty et al. 1999; Shanmugam et al. 2015). For example, Researchs show that Neoprzewaquinone A (NEO) inhibits TNBC cell migration mainly by targeting PIM1 and inhibiting STAT3 signaling (Zhao et al. 2023). β-elemonic acid inhibits growth of human castration-resistant prostate cancer cells through the suppression of JAK2/STAT3/MCL-1 signal pathways (Bao et al. 2021). TIMP-1 mediates the inhibitory effect of interleukin-6 on the proliferation of a hepatocarcinoma cell line in a STAT3-dependent manner (Guo et al. 2007). In addition, other studies have shown that changes in downstream target genes mediated by STAT3 are closely related to cancer proliferation and metastasis.During the process of tumor cell metastasis, STAT3 can regulate the expression of ZEB1, SNAIL, and TWIST, and it is involved in the process of EMT (Xiong et al. 2012; Liu et al. 2021; Kim et al. 2014, 2019). The overexpression of estrogen-related receptor alpha (ERR-α) by STAT3 promotes EMT in TNBC via reducing E-cadherin and increasing ZEB1 and N-cadherin (Ma et al. 2019). Twist is another EMT transcriptional factor, and the interaction between STAT3 and Twist can affect tumor metastasis. For example, MEST induces Twist-1-mediated EMT via STAT3 activation in breast cancers (Kim et al. 2019). In this article, our experimental data showed that knockdown of POC1A increases mRNA and protein of CDH1 but decreases mRNA and protein of CDH2, ZEB1, SNAIL1, SNAIL2 and VIMENTIN. We speculate that POC1A may promote STAT3 phosphorylation, After STAT3 phosphorylation, it enters the nucleus and activates ZEB1/SNAIL, thereby inhibiting CDH1 and promoting the expression of CDH2/Vimentin(Wendt et al. 2014; Mu et al. 2019; Lee et al. 2024).

The activation of the STAT3 pathway could promote proliferation, invasion, metastasis, and angiogenesis, while inhibiting apoptosis in breast cancer cells (Kamran et al. 2013). Therefore, targeting the STAT3 pathway may be a therapeutic method for breast cancer. 

**Fig. 8:**
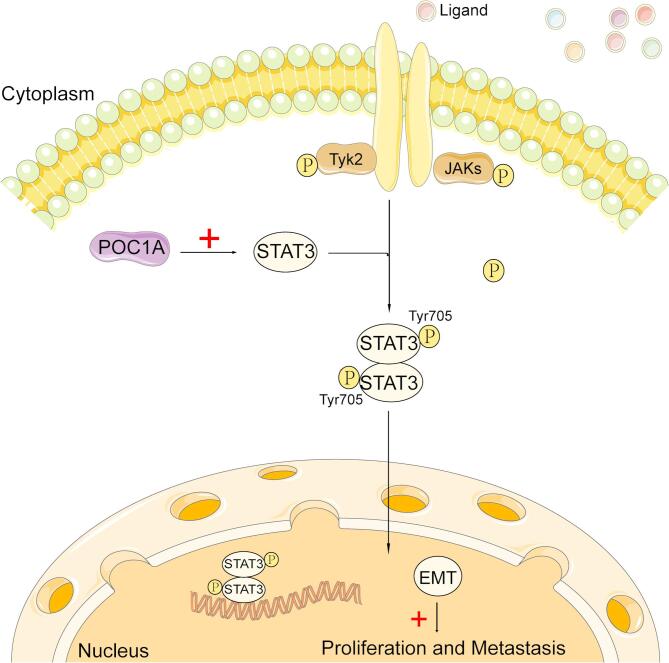
POC1A Induces Epithelial–Mesenchymal Transition to Promote growth and Metastasis through the STAT3 Signaling Pathway in Triple-negative Breast Cancer

Based on the above research, we found that POC1A can regulate the EMT of TNBC via the STAT3 pathway, promoting the migration and invasion of TNBC (Fig. 8). Therefore, it can serve as a new biomarker and provide valuable insights for the treatment of TNBC. However, this study has certain limitations and only provides preliminary evidence that knocking down POC1A leads to a decrease in P-STAT3 expression and changes in cell phenotype. However, rescue experiments were not conducted in vivo and at the cellular level to further prove that POC1A can better support POC1A to regulate the proliferation, migration and invasion of breast cancer by affecting STAT3 phosphorylation directly. In the future, we will conduct relevant research in this area to improve our experiments.

## Conclusion

Our study has revealed that POC1A is significantly upregulated in TNBC, and it is related to poor patient survival. Notably, this study is the first to demonstrate that POC1A knockdown could inhibit proliferation and migration with respect to the EMT of TNBC cell lines by modulating STAT3 signaling pathways. Thus, our paper reveals a novel mechanism: POC1A induces EMT via STAT3 pathways with respect to the development of TNBC. While our study provides significant insight into TNBC metastasis, it is vital to recognize certain deficiencies. Therefore, more studies are needed to elucidate the regulatory mechanism by which POC1A influences the JAK-STAT3 pathway in TNBC. Although the further mechanism of POC1A in coordinating STAT3 pathway is unclear, our results suggest that POC1A may be an important regulator of STAT3 mediated proliferation and metastasis of TNBC. Therefore, based on the above research, we speculate that blockade of the pathways POC1A expression or inhibition of the interaction between POC1A and its receptor may provide a new strategy for the treatment of TNBC.

## Supplementary Information

Supplementary Material 1: Supplementary Figure 1. Kaplan‑Meier analysis of the results showed that POC1A gene expression was related to the of patients OS, DMFS and RFS with breast cancer; patients with a high POC1A expression had a poor prognosis; patients with a low POC1A expression had an improved prognosis. *P* values were calculated using Pearson correlation analysis. Abbreviation: OS: overall survival; DMFS: distant metastasis-free survival; RFS: recurrence free survival.

Supplementary Material 2: Supplementary Figure 2. A. 4T1 cells were transfected with lentiviral vectors encoding Poc1a short hairpin RNA vectoror scramble vector. RT-qPCR and Western blotting detected the expression of 4T1-Poc1a cells. B-C. Cell proliferation and migration assessed by CCK-8 assay and wound healing assay. All the experiments were repeated three times with similar results independently. Data represent mean ± SD. *P* values were calculated using unpaired two-tailed Student’s t tests. Abbreviation: *, *p*<0.05; **, *p*<0.01; ***, *p*<0.001.

Supplementary Material 3: Supplementary Figure 3. A. quantization of figure 2B WBs. B. quantization of figure 2D WBs. C. quantization of figure 3D WBs. D. quantization of figure 4C WBs. E. quantization of figure 5D WBs. F. quantization of figure 6C WBs. G. quantization of Supplementary Figure 2A WBs. All the experiments were repeated three times with similar results independently. Data represent mean ± SD. *P* values were calculated using unpaired two-tailed Student’s t tests. Abbreviation: *, *p*<0.05; **, *p*<0.01; ***, *p*<0.001.

Supplementary Material 4: Supplementary-Table S1.

Supplementary Material 5: Supplementary-Table S2.

## Data Availability

No datasets were generated or analysed during the current study.

## Abbreviations

TNBC: Triple-negative breast cancer
IHC: Immunohistochemistry
RT-qPCR: Reverse Transcription-quantitative Polymerase Chain Reaction
IF: Immunofluorescence
EMT: Epithelial–mesenchymal transition
TGF: Transforming growth factor
BRCA: Breast cancer
