# Correction: POC1A induces epithelial–mesenchymal transition to promote growth and metastasis through the STAT3 signaling pathway in triple-negative breast cancer

**DOI:** 10.1186/s10020-025-01369-1

**Published:** 2025-11-03

**Authors:** Yuzhou Qian, Yu Che, Shanqi Li, Xue Zhang, Qingshu Li, Yong Zhu, Long Wang, Xuedong Yin

**Affiliations:** 1 Institute of Life Sciences, Chongqing Medical University, Chongqing, 400016 China; 2 Department of Breast and Thyroid Surgery, Chongqing Key Laboratory of Molecular Oncology and Epigenetics, The First Affiliated Hospital of Chongqing Medical University, Chongqing, 400016 China; 3 Department Of Breast Cancer Center, Chongqing University Cancer Hospital, Chongqing, 400030 China; 4 Department of Respiratory Medicine, The First Affiliated Hospital of Chongqing Medical University, Chongqing, 400016 China; 5 Department of Pathology, College of Basic Medicine, Chongqing Medical University, Chongqing, 400016 China; 6 Molecular Medicine Diagnostic and Testing Center, Chongqing Medical University, Chongqing, 400016 China; 7 Department of Pathology, the First Affiliated Hospital of Chongqing Medical University, Chongqing, 400016 China

**Correction: Mol Med 31, 280 (2025)**

**https://doi.org/10.1186/s10020-025-01315-1**

In this article (Qian et al. 2025), the wrong Supplementary table S1 and table 1 was originally published with this article; it has now been replaced with the correct tables.

The original article has been corrected.

Incorrect Table

**Table 1:** clinicopathological features of TCGA triple-negative breast cancer patients

**Characteristic**	**Number of Cases**	**POC1A**		
		**High (n)**	**Low (n)**	***P*** **-value**
Age				
> 50	102	44	58	0.04*
≤ 50	66	40	26	
Tumor Size				
T1	26	11	15	0.1434
T2	58	27	31	
T3	9	7	2	
T4	2	0	2	
Pathologic stage				
I	25	9	16	0.0174*
II	105	61	44	
III	33	12	21	
IV	3	0	3	
T				
T1-2	140	72	68	0.4198
T3-4	27	11	16	
N				
N0	97	44	53	0.005*
N1	45	32	13	
N2	18	6	12	
N3	8	2	6	
M				
M0	142	70	72	0.6513
M1	4	1	3	

*Represent statistically significant

Correct Table

**Table 1:** Clinicopathological features of TCGA triple-negative breast cancer patients

Characteristic	Number of Cases	POC1A		
		High (n)	Low (n)	*P*-value
Age				
> 50	102	44	58	0.04*
≤ 50	66	40	26	
Pathologic stage				
I	25	9	16	0.0174*
II	105	61	44	
III	33	12	21	
IV	3	0	3	
Unknown	2	2	0	
Tumor size				
T1	42	20	22	0.6416
T2	98	52	46	
T3	18	8	10	
T4	9	3	6	
Unknown	1	1	0	
Lymph node metastasis				
N0	97	44	53	0.005*
N1	45	32	13	
N2	18	6	12	
N3	8	2	6	
Metastasis				
M0	142	70	72	0.6513
M1	4	1	3	
Unknown	22	13	9	

^*^Represent statistically significant

## Supplementary Information

Supplementary Material 1: Supplementary Table 1. The sequences of shRNA and primers for real-time PCR assays
