# Supplementary figures and images for: POC1A induces epithelial–mesenchymal transition to promote growth and metastasis through the STAT3 signaling pathway in triple-negative breast cancer
Source: Mol Med. 2025 Aug 19;31:280. doi: 10.1186/s10020-025-01315-1 (PMC12366406; doi:10.1186/s10020-025-01315-1)

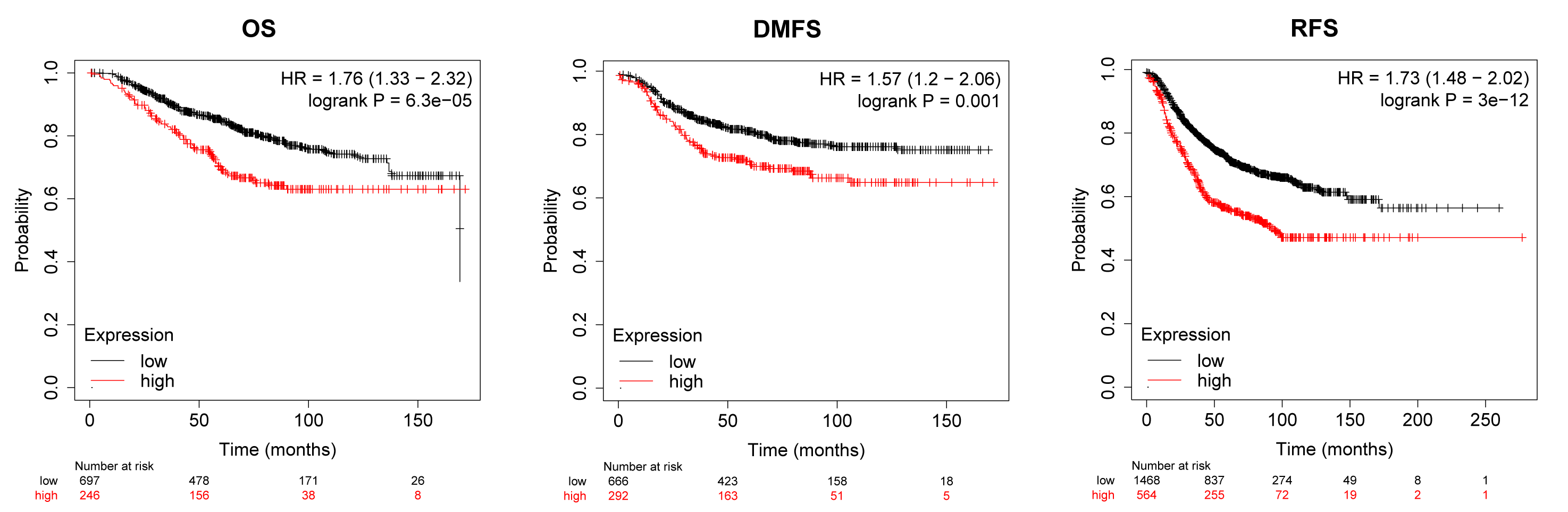

Supplement: Supplementary file 1 — Supplementary Material 1: Supplementary Figure 1. Kaplan‑Meier analysis of the results showed that POC1A gene expression was related to the of patients OS, DMFS and RFS with breast cancer; patients with a high POC1A expression had a poor prognosis; patients with a low POC1A expression had an improved prognosis. P values were calculated using Pearson correlation analysis. Abbreviation: OS: overall survival; DMFS: distant metastasis-free survival; RFS: recurrence free survival. [file 10020_2025_1315_MOESM1_ESM.tif]

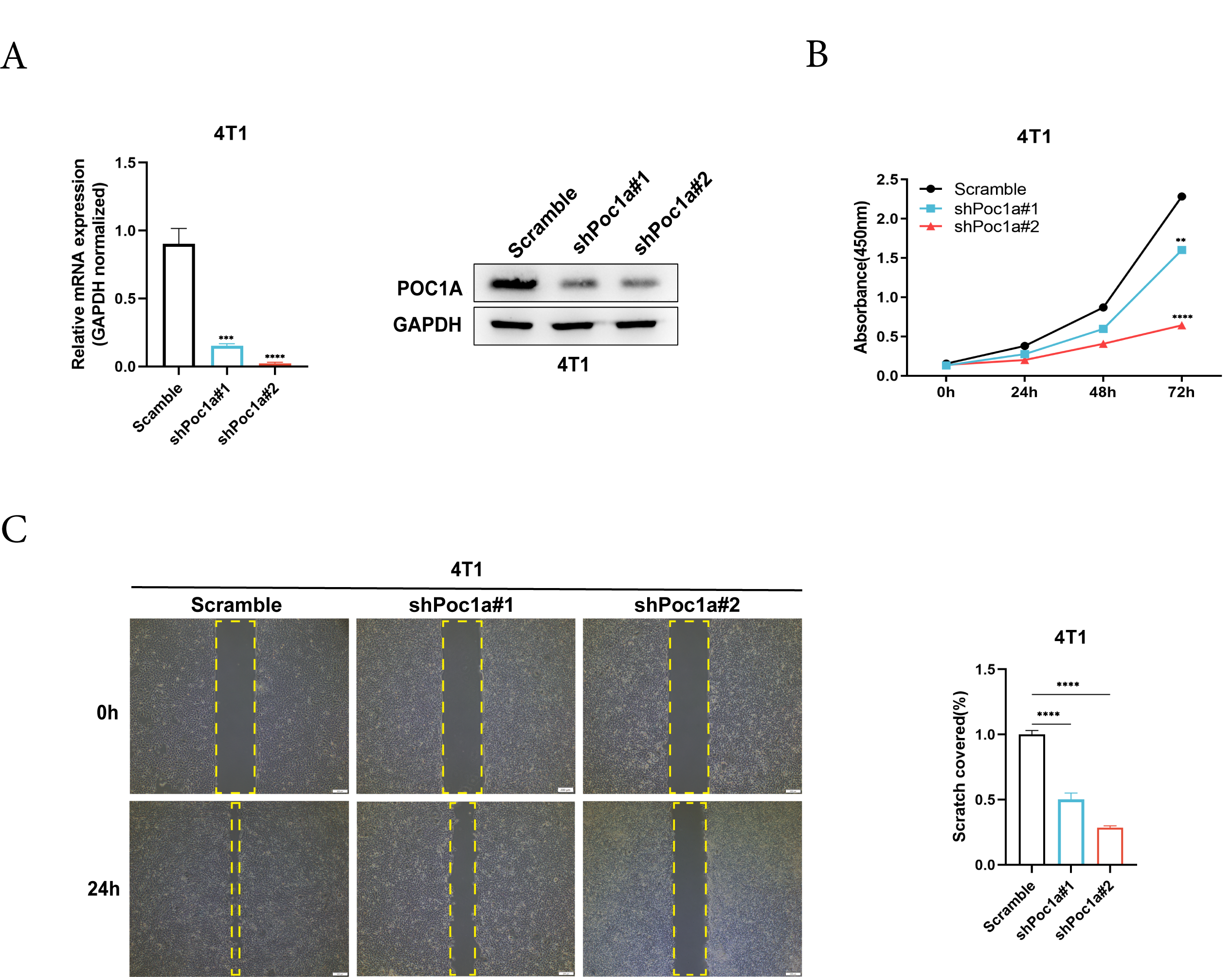

Supplement: Supplementary file 2 — Supplementary Material 2: Supplementary Figure 2. A. 4T1 cells were transfected with lentiviral vectors encoding Poc1a short hairpin RNA vectoror scramble vector. RT-qPCR and Western blotting detected the expression of 4T1-Poc1a cells. B-C. Cell proliferation and migration assessed by CCK-8 assay and wound healing assay. All the experiments were repeated three times with similar results independently. Data represent mean ± SD. P values were calculated using unpaired two-tailed Student’s t tests. Abbreviation: *, p<0.05; **, p<0.01; ***, p<0.001. [file 10020_2025_1315_MOESM2_ESM.tif]

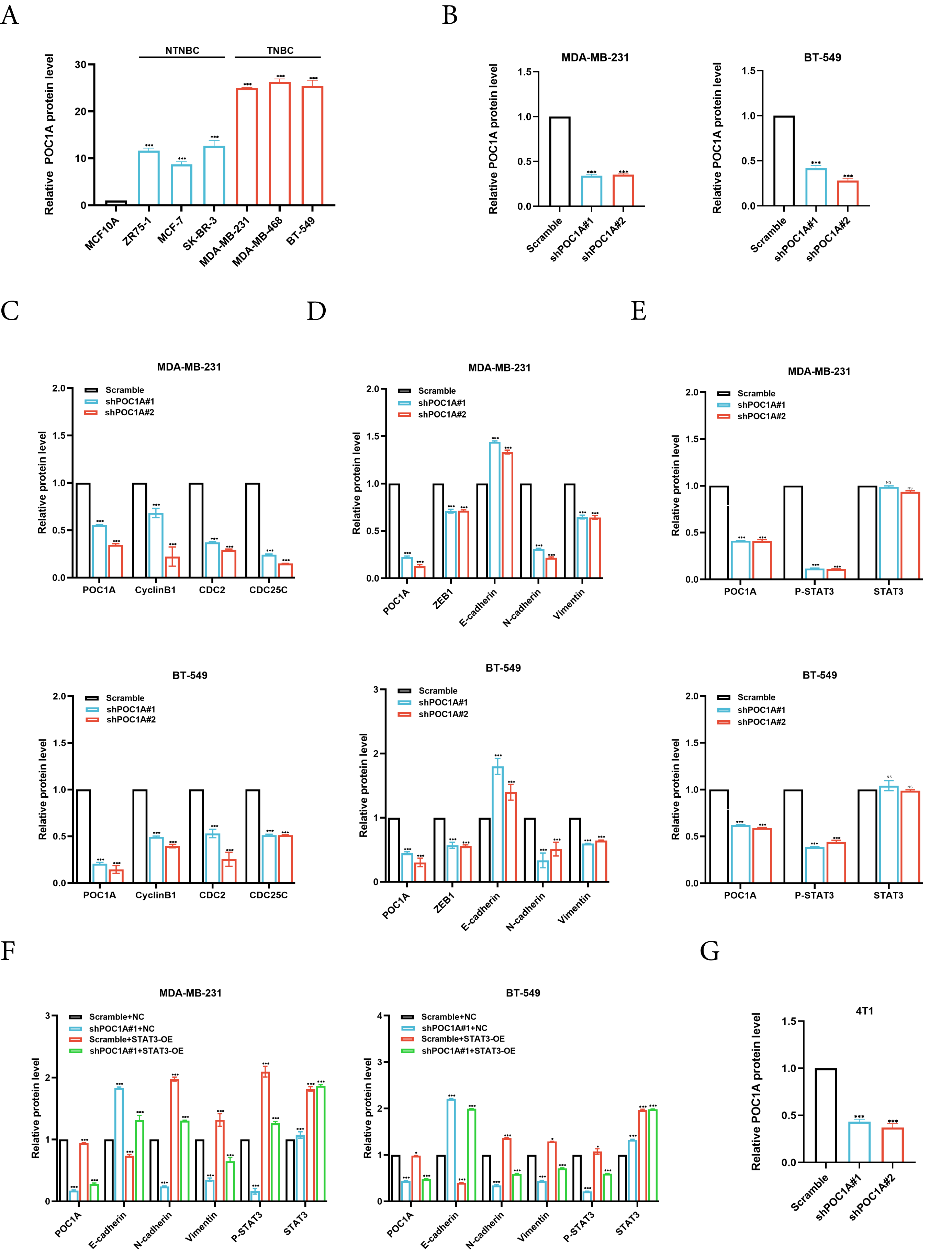

Supplement: Supplementary file 3 — Supplementary Material 3: Supplementary Figure 3. A. quantization of figure 2B WBs. B. quantization of figure 2D WBs. C. quantization of figure 3D WBs. D. quantization of figure 4C WBs. E. quantization of figure 5D WBs. F. quantization of figure 6C WBs. G. quantization of Supplementary Figure 2A WBs. All the experiments were repeated three times with similar results independently. Data represent mean ± SD. P values were calculated using unpaired two-tailed Student’s t tests. Abbreviation: *, p<0.05; **, p<0.01; ***, p<0.001. [file 10020_2025_1315_MOESM3_ESM.tif]
