# Supplementary material for: POC1A induces epithelial–mesenchymal transition to promote growth and metastasis through the STAT3 signaling pathway in triple-negative breast cancer
Source: Mol Med. 2025 Aug 19;31:280. doi: 10.1186/s10020-025-01315-1 (PMC12366406; doi:10.1186/s10020-025-01315-1)
Supplement: Supplementary file 4 — Supplementary Material 4: Supplementary-Table S1. [file 10020_2025_1315_MOESM4_ESM.docx]

**Supplementary Table 1**. The sequences of shRNA and primers for real-time PCR assays.

| **Name** | **Catalog number** |
| --- | --- |
| shPOC1A#1 | CAGTGATGACAAGACTGTTAA |
| shPOC1A#2 | GGCAACTCATCGCCAGAAA |
| shPoc1a#1 | GGGAATGTATCCACTCATA |
| shPoc1a#2 | GCGACTGACACTAACAGAA |
| GAPDH-F | AAGGTGAAGGTCGGAGTCAAC |
| GAPDH-R | GAAGGGGTCATTGATGGCAAC |
| POC1A-F | GTACCCAATGTCAAAGGTGAGTC |
| POC1A-R | CCAGTTGATATGCTGGCTCAG |
| CDH1-F | CGAGAGCTACACGTTCACGG |
| CDH1-R | GGGTGTCGAGGGAAAAATAGG |
| CDH2-F | TGCGGTACAGTGTAACTGGG |
| CDH2-R | GAAACCGGGCTATCTGCTCG |
| ZEB1-F | GATGATGAATGCGAGTCAGATGC |
| ZEB1-R | ACAGCAGTGTCTTGTTGTTGT |
| SNAIL1-F | TCGGAAGCCTAACTACAGCGA |
| SNAIL1-R | AGATGAGCATTGGCAGCGAG |
| SNAIL2-F | CGAACTGGACACACATACAGTG |
| SNAIL2-R | CTGAGGATCTCTGGTTGTGGT |
| Vimentin-F | AGTCCACTGAGTACCGGAGAC |
| Vimentin-R | CATTTCACGCATCTGGCGTTC |
| PIM1-F | GGCTCGGTCTACTCAGGCA |
| PIM1-R | GGAAATCCGGTCCTTCTCCAC |
| MCL1-F | GGAGATTCCTGACCAGAACATTG |
| MCL1-R | CGACTGGGCTTTATCAAGACAT |
| TIMP1-F | CTTCTGCAATTCCGACCTCGT |
| TIMP1-R | ACGCTGGTATAAGGTGGTCTG |
| TIMP2-F | ACACGCAATGAAACCGAAGC |
| TIMP2-R | ACTTGGGGGTCAGGAGTCTT |
| Gapdh-F | AGGTCGGTGTGAACGGATTTG |
| Gapdh-R | TGTAGACCATGTAGTTGAGGTCA |
| Poc1a-F | CTCCCCATCGGGACACCTT |
| Poc1a-R | GTTGACCACACTTTGACTGTCTT |
