# Supplementary material for: POC1A induces epithelial–mesenchymal transition to promote growth and metastasis through the STAT3 signaling pathway in triple-negative breast cancer
Source: Mol Med. 2025 Aug 19;31:280. doi: 10.1186/s10020-025-01315-1 (PMC12366406; doi:10.1186/s10020-025-01315-1)
Supplement: Supplementary file 5 — Supplementary Material 5: Supplementary-Table S2. [file 10020_2025_1315_MOESM5_ESM.docx]

**Supplementary Table 2**. The antibodies list used in this study.

| **Name** | **Source** | **Catalog number** |
| --- | --- | --- |
| GAPDH | Proteintech | 10494-1-AP |
| POC1A | Thermo Fisher | PA5-110190 |
| CDC25C | Cell Signaling Technology | 4688T |
| CDC2 | Cell Signaling Technology | 9116T |
| Cyclin B1 | Cell Signaling Technology | 12231T |
| ZEB1 | Cell Signaling Technology | 70512T |
| E-cadherin | Cell Signaling Technology | 3195 |
| N-cadherin | Cell Signaling Technology | 13116T |
| Vimentin | Cell Signaling Technology | 5741T |
| P-STAT3 (Tyr705) | Cell Signaling Technology | 9145 |
| STAT3 | Cell Signaling Technolog | 9139 |
